# Sensory Analysis in Assessing the Possibility of Using Ethanol Extracts of Spices to Develop New Meat Products

**DOI:** 10.3390/foods9020209

**Published:** 2020-02-18

**Authors:** Krystyna Szymandera-Buszka, Katarzyna Waszkowiak, Anna Jędrusek-Golińska, Marzanna Hęś

**Affiliations:** Department of Gastronomy Science and Functional Foods, Faculty of Food Science and Nutrition, Poznan University of Life Sciences, Wojska Polskiego 31, 60-624 Poznan, Poland; krystyna.szymandera_buszka@up.poznan.pl (K.S.-B.); anna.jedrusek-golinska@up.poznan.pl (A.J.-G.); marzanna.hes@up.poznan.pl (M.H.)

**Keywords:** new product development, consumer research, sensory profiling, meat products, ethanol extracts of spices

## Abstract

The food industry has endeavoured to move toward the direction of clean labelling. Therefore, replacing synthetic preservatives with natural plant extracts has gained significant importance. It is necessary to determine whether products enriched with such extracts are still accepted by consumers. In this study, consumer tests (n = 246) and sensory profiling were used to assess the impact of ethanol extracts of spices (lovage, marjoram, thyme, oregano, rosemary, and basil; concentration 0.05%) on the sensory quality of pork meatballs and hamburgers. The desirability of meat products with spice extracts to consumers depended on the added extract. The highest scores were for products with lovage extract, whose sensory profile was the most similar to the control sample without the addition of an extract (with higher intensity of broth taste compared with the others). Products with rosemary and thyme extracts were characterised by lower desirability than the control. This was related to the high intensity of spicy and essential oil tastes, as well as the bitter taste in the case of products with thyme. The studied extracts of spices allow for the creation of meat products (meatballs and hamburgers) with high consumer desirability, however, the high intensity of essential oil and spicy tastes might be a limitation.

## 1. Introduction

Lipid oxidation is one of the main factors resulting in undesirable changes in food. The oxidation of meat lipids, taking place during processing and storage, leads to a considerable deterioration of processed meat quality [1]. As a result of fatty acid oxidation (especially polyenic acids), various products are formed, both volatile and non-volatile [2]. These oxidation products deteriorate physico-chemical properties and affect sensory attributes and the health quality of meat products [3,4]. For this reason, the food industry is interested in the development of novel food products enriched with natural antioxidants [5]. The development of food additives, which are extracts of natural compounds exhibiting antioxidant and antimicrobial activity, is a crucial step toward the production of food with health benefits [6,7]. A source of those antioxidant compounds can be spice plants, such as rosemary (*Rosmarinus officinalis* L.), thyme (*Thymus vulgaris* L.), marjoram (*Origanum majorana* L.), oregano (*Origanum vulgare* L.), basil (*Ocimum basilicum* L.), and lovage (*Levisticum officinale*) [8,9,10], which are more and more frequently used in the food industry. The results of earlier studies indicated that phenolic compounds from the spices (their ethanol extracts) show strong antioxidant activities both in model systems and food [10,11,12,13,14]. These ethanol extracts also showed antibacterial activity [15,16,17]. In the previous studies, their effectiveness was assessed in various differently processed meat products. Naveena et al. [18] investigated the effect of oil soluble and water dispersible carnosic acid extract from dried rosemary leaves at two different concentrations (0.0023% and 0.013%) on the inhibition of oxidation in raw and cooked ground buffalo meat patties. It was reported that carnosic acid extract reduced the TBARS (thiobarbituric acid reactive substances) content by 39%–47% at a lower concentration and by 86%–96% at a higher concentration in cooked buffalo meat patties compared to controls. The extracts also inhibited peroxide value and free fatty acid formation in the products. Moreover, the extract added at the higher concentrations stabilised the colour of raw buffalo meat. Sebranek et al. [19] tested the commercial rosemary extract at concentrations of 0.15% and 0.25% in frozen and precooked-frozen pork sausage, and between 0.05% and 0.3% in refrigerated fresh pork sausage. They reported that the rosemary extract added to refrigerated sausage at 0.25% showed similar antioxidant activity to synthetic antioxidants (BHA/BHT). Bilska et al. [20] added 0.05% of rosemary extract to enhance the oxidative stability of pork liver pâté with fat replaced by 20% of flaxseed oil and showed that the addition significantly slowed down lipid oxidation during the storage of the pâté. Oregano extract (0.02%) has been shown to inhibit lipid oxidation in cooked ground beef and pork (30% fat, fresh meat basis) [21,22] and in lean raw beef [23]. Rojas and Brewer [22] studied the effects of water-soluble oregano extract in cooked beef and pork. They found that the extract added at 0.02% was effective at reducing lipid oxidation in vacuum-packaged cooked beef samples stored at −18 °C for 4 months. Trindade et al. [24] studied the effect of the addition of rosemary and oregano extracts (0.04%) on the sensory qualities of irradiated (7 kGy) beef burger during frozen storage, and it was concluded that these natural antioxidants could prevent lipid oxidation without affecting the sensory scores of treated meat samples. The similar effect was observed by Manhani et al. [25] in the case of the addition of rosemary and oregano commercial extracts (0.04%) to precooked beef hamburgers. Gramatina et al. [17] investigate the potential use of ethanol extracts from selected herbals to maintain the pork meat quality during refrigerated storage and they found that oregano and lovage extracts inhibited microbial growth after soaking in the extracts, thus extending the shelf-life of pork meat. Thyme, marjoram, and basil were also studied as antioxidants in meat products. El-Alim et al. [26] investigated the use of ground spices and spice extracts (including marjoram, basil and thyme) as antioxidants in raw ground chicken and ground pork. Authors reported that TBARS formation was inhibited in refrigerated and frozen samples of ground chicken which were treated with 10 g dried spices/kg. They also examined the ethanol extracts of basil, sage, thyme, and ginger (prepared in the laboratory) as antioxidants in ground pork (ground pork treated with the extracts at a concentration of 1 mL/10 g) [26], and found that sage, thyme, and basil extracts were more effective at inhibiting TBARS values in ground pork than ginger extract. Hęś et al. [12] investigated the effect of rosemary, green tea, and thyme extracts (0.05%) on lipid stability and the protein nutritional value of frozen-stored fried balls from ground pork and found that the additions limited lipid oxidation, as well as reduced changes in methionine and lysine content, and protein digestibility compared to the control. Sarıçoban and Yilmaz [27] showed that the addition of thyme essential oils at 0.05% decreased oxidation and microbial grow, extending the shelf-life of chicken pâté.

Adding the extracts into product formulations may allow developing new processed meats and culinary products with a longer shelf-life and high nutritional value [3,12,28,29]. Additionally, some of the health effects attributed to the antioxidant, antimicrobial, and anti-inflammatory effects of phenolic compounds present in the extracts indicate their potential protection against cardiovascular disease, neurodegeneration, type 2 diabetes, and cancer [30,31,32,33].

In the process of food product development, there are two aspects that are necessary to determine, apart from studies on antioxidant activity of plant extracts during production and storage of meat products. These aspects are whether products enriched with such extracts still show good sensory quality and whether they are accepted by consumers. In technological research, a new product is first created and then its nutritional value, technological features, shelf life, etc., are examined and, finally, its sensory quality is determined. On the contrary, in the sensory approach to designing new products, the sensory quality of the designed products is checked in detail first to know which product attributes are worth strengthening or masking. In this case, it is vital to combine the results of consumer assessment (in which consumer preferences are assessed and thus their subjective feelings) with the results of objective methods (e.g., Quantitative Descriptive Analysis) [34]. By comparing the results of both methods, it is possible to obtain full information concerning sensory quality and to determine which product attributes and their intensities are accepted by consumers [34,35]. Only tasty food has a chance of being bought and consumed.

Therefore, the aim of this study was the application of consumer tests and the sensory profiling method to assess the impact of ethanol extracts of selected spices (rosemary, thyme, marjoram, oregano, basil, and lovage) on the sensory quality of pork meatballs and hamburgers.

## 2. Materials and Methods

The pork meatballs and hamburgers enriched with ethanol extracts from the selected spice (rosemary, thyme, marjoram, oregano, basil, and lovage) were the research material of this study.

The rosemary extract (Oxy’Less U; powder form, dry extract: 95 ± 3%) was purchased from Naturex (Avignon, France). It was an unrefined rosemary extract obtained by ethanol extraction of rosemary leaves (the commercial characteristic based on the producer information). The other extracts were prepared according to the procedure described by Hęś and Gramza-Michałowska [12]. Briefly, dried leaves of thyme (*Thymus vulgaris* L.), marjoram (*Origanum majorana* L.), oregano (*Origanum vulgare* L.), basil (*Ocimum basilicum* L.), and lovage (*Levisticum officinale*) were purchased from a local herb shop, which guaranteed the origin and the freshness of raw materials. Dried materials (100 g) were macerated with 250 mL of 80% ethanol overnight at room temperature. The suspension was filtered, the residue was mixed with another portion of ethanol, and the procedure was repeated three times. The extracts were collected, and ethanol was removed at 50 °C using a rotary vacuum evaporator (Buchi, Flawil, Switzerland). Then, the extracts were freeze-dried (Alpha 1–4 LSC Freeze dryer, Christ, Osterode am Harz, Germany). The dried extracts were stored at 4 °C in a dark place. The average total phenolic contents of the ethanol extracts (expressed as mg gallic acid per g of dry mass) are as follows: 161.3 mg rosemary extract, 229.6 mg thyme extract, 105.5 mg oregano extract, 36.4 mg lovage extract, 113.2 mg marjoram extract, and 129.6 mg basil extract [12,36] and unpublished results.

For pork meatball and hamburger preparation, pork (from the best end of the neck) was minced (mesh size of 3mm) and mixed thoroughly (approx. 10 min) with the other ingredients using a homogenizer (Foss, Hilleroed, Denmark), producing batter containing 70% meat, 15.8% water, 8% breadcrumbs, 5% eggs, 1% salt, and 0.2% pepper. The batter was divided into seven portions. One was the control sample, and ethanol extracts were added to the others: rosemary, thyme, marjoram, oregano, basil, and lovage (0.05% of meat batter). The addition of the extracts was selected based on our previous study concerning the protective effect of the extracts against oxidation [12,20,28] and EU Regulations [37]. For heat-treated meat products, the EU Regulations No 723/2013 [37] set the maximum addition of rosemary extract at 15 mg/kg. Our previously published studies reported [12,20] that 0.05% additives of rosemary and thyme extracts significantly slowed down lipid oxidation during storage of meat products. This observation was supported by the studies of other scientists who have shown the protective effect of rosemary [19,24,25], thyme [27], and oregano [21,22,23,24,25] extracts added at a similar level to meat products (that is 0.02%–0.05%). Therefore, the 0.05% addition was used in the case of all tested extracts to compare the impact of the extracts on the sensory quality of meatballs and hamburgers.

Round samples of a similar weight (50 g ± 1 g) were formed. Next, the samples were roasted (hot air at 160 °C for 8 min) to prepare the hamburgers, or steamed (100 °C for 16 min) to prepare the meatballs, in a convection oven (Rational, Landsberg am Lech, Germany). The products were prepared (on a laboratory scale) in the technology laboratory located at the Faculty of Food Science and Nutrition, Poznań University of Life Sciences (Poland).

Sensory tests were conducted in a sensory analysis laboratory equipped with individual booths (at a controlled temperature of 21 ± 1 °C and combined natural/artificial light) designed according to ISO standards [38]. The laboratory was located at the Faculty of Food Science and Nutrition, Poznań University of Life Sciences (Poland). The tests were carried out in 2018 between 9 a.m. and 3 p.m. The samples of the products were evaluated when warm, on the same day they were made, and were served on odourless white plates. The samples were coded with random three-digit numbers, and the serving order of samples was random (program Analsens was used for coding and arrangement of serving order). Water was used to cleanse the mouth between samples. No ethical approval was required for this study. Participants were informed about the study’s aim and that their participation was entirely voluntary, so that they could stop the analysis at any point and the responses would be anonymous.

For detailed sensory characteristics of hamburgers and meatballs with spice ethanol extracts, the quantitative descriptive analysis was applied, i.e., sensory profiling. The analysis was conducted by an 8-member trained panel [39]. A total of 13 descriptors, elementary attributes of aroma (meat, peppery, fried, spicy, bitter, and essential oil aromas) and taste (salty, broth, fried, spicy, bitter, and essential oil tastes) of tested meat products were evaluated. These descriptors of aroma and taste were selected in preliminary tests. The intensity of each score was determined using a 10-cm linear scale with appropriate margin descriptions. For attributes of aroma and taste, uniform margin denotations were applied: “undetectable”–“very intensive”. Four samples were served during a session to the panellists (three samples with spice extracts and control sample in one session); two sessions were carried out in one day. All samples were assessed in two independent replications.

Simultaneously with the profile analysis of meats products, the consumer test was conducted among a group of 246 people who consume meat products at least once every week, aged 20–50. They were neither students, professors, nor administrative employees of Poznań University of Life Sciences; 53% of the participants were women. Consumers evaluated the aroma, taste, and overall desirability of the meat products. For the consumer test, a 10 cm hedonic graphic scale was applied with the following margin denotations: “undesirable”–“highly desirable”. All consumers evaluated both meatballs and hamburgers in one session (order of serving: seven samples, a break of 0.5 h, and seven samples).

Statistical analyses were conducted using Statistica (v.13.1, StatSoft Tulsa, OK, USA). The effects of spice ethanol extracts (L = 7; six selected spice extracts and control sample with no extract) were analysed. The results of sensory tests were subjected to the analysis of variance (ANOVA), and then post hoc Tukey’s test was applied at a significance level of *p <* 0.05 to compare the means. Hierarchical Cluster Analysis (HCA) was performed to identify similar groups of meat products based on consumer test results (i.e., aroma, taste, and overall consumer desirability). Ward’s method was used. According to the method, the means for all variables are calculated for each cluster. For each case, the squared Euclidean distance to the cluster means is calculated; the two clusters that merge are those that result in the smallest increase in the overall sum of the squared within-cluster distances [40]. Principal Component Analysis (PCA) was applied to the data sets from the sensory profiling of products to assess differences and similarities in sensory profiles based on their aroma and taste descriptors.

## 3. Results

### 3.1. Consumer Test Results

The results of the consumer evaluation showed that pork meatballs and hamburgers with ethanol extracts of spices were characterised by varied overall desirability (Table 1, one-way ANOVA, *p* < 0.05). Both meat products with lovage extract were characterised by the significantly higher overall consumer desirability compared to the products with rosemary extract and thyme extract. The same was observed in the case of these products’ aroma and taste assessment. Moreover, the consumer desirability of aroma and taste of the hamburgers and meatballs with lovage extract were significantly higher than those with basil extract and oregano extract.

**Figure 1:**
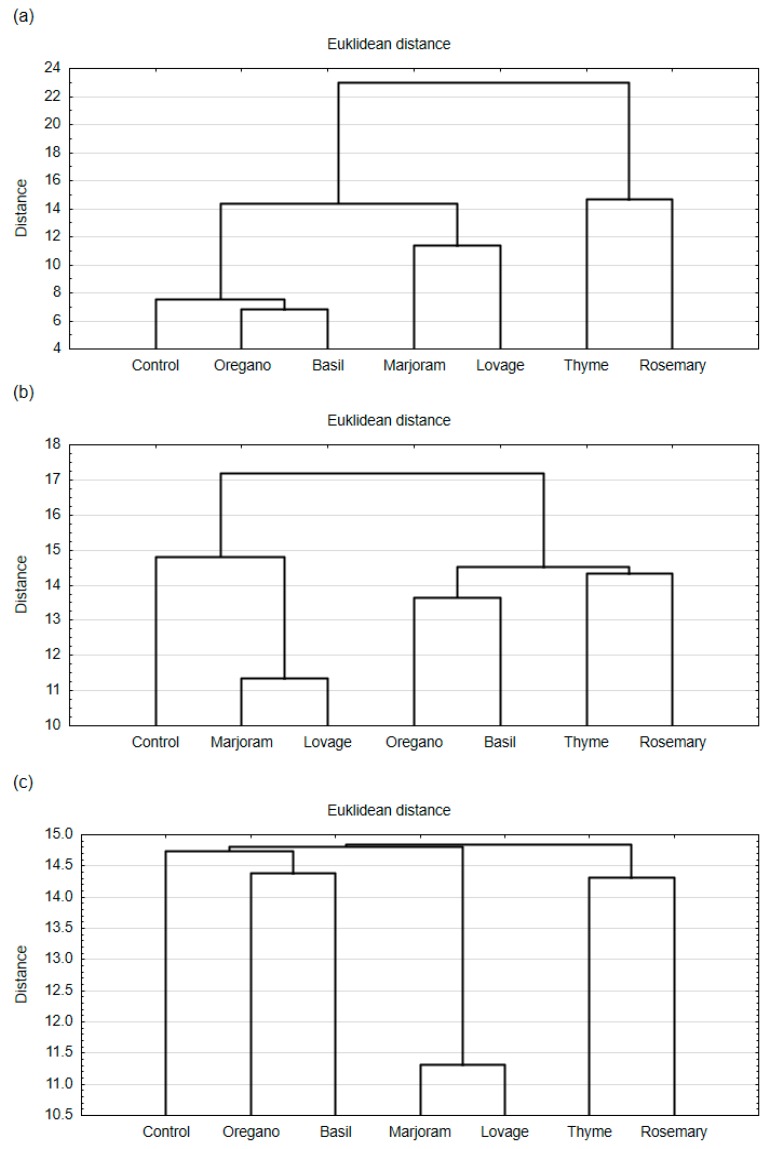
Dendrograms of hierarchical cluster analysis (HCA) for (**a**) the desirability of the aroma, (**b**) the desirability of the taste, and (**c**) the overall desirability of the pork meatballs with ethanol extracts of the spices (rosemary, thyme, lovage, marjoram, basil, oregano; 0.05%) and the control sample (without any extract) to the consumers.

**Figure 2:**
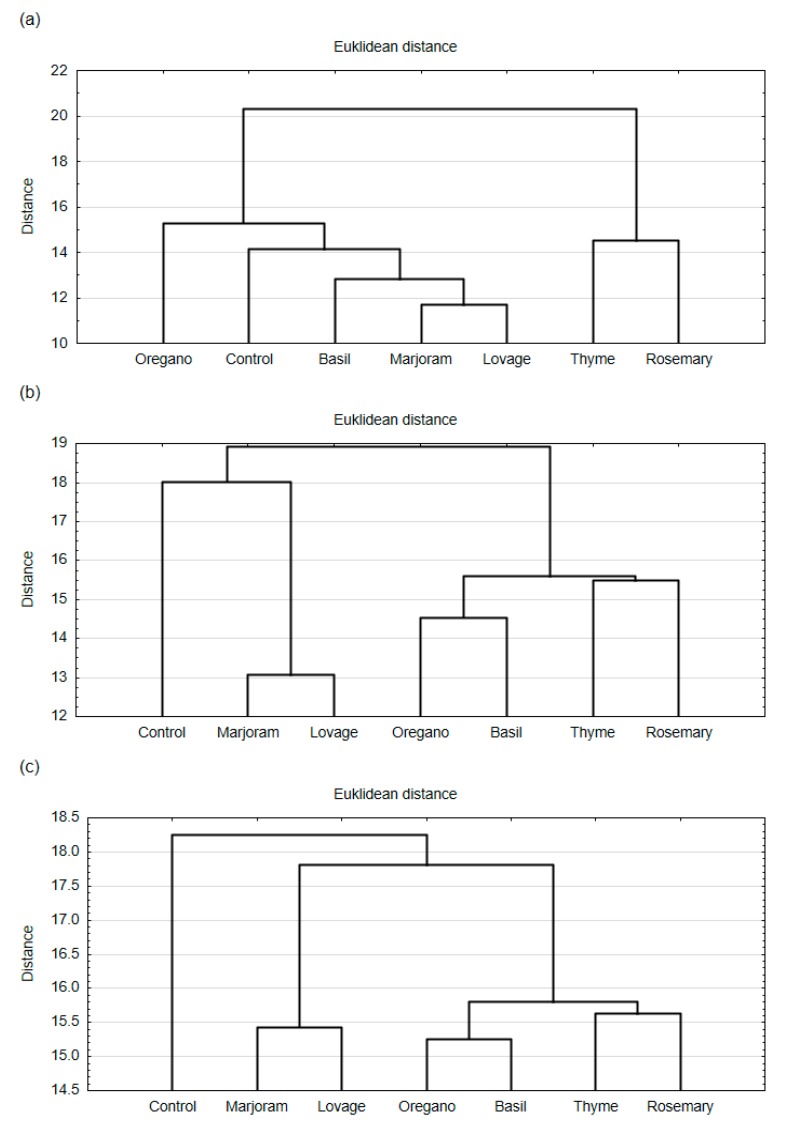
Dendrograms of hierarchical cluster analysis (HCA) for (**a**) the desirability of the aroma, (**b**) the desirability of the taste, and (**c**) the overall desirability of the pork hamburgers with ethanol extracts of the spices (rosemary, thyme, lovage, marjoram, basil, oregano; 0.05%) and the control sample (without any extract) to the consumers.

The hierarchical cluster analysis (HCA) grouped the meat products (meatballs and hamburgers) into clusters based on the similarity of overall consumer desirability and desirability of aroma and taste. HCA results are presented as dendrograms (Figure 1 and Figure 2).

The results of statistical analysis showed that the addition of spice extract influenced the aroma and taste desirability of both meat products (Table 1; one-way ANOVA, *p* < 0.05). In the case of meatballs and hamburgers, HCA analysis showed (Figure 1a and Figure 2a) that consumers distinguished the aroma of the samples with thyme extract and rosemary extract from the other samples (they formed a separate cluster). A similarity was found between meat products with marjoram extract and lovage extract (they were arranged in one cluster based on their aroma desirability). Based on the results of taste desirability, HCA analysis showed a different arrangement of the tested products in clusters when compared to aroma desirability (Figure 1b and Figure 2b). The taste desirability of samples with lovage extract and samples with marjoram extract was the closest (they formed clusters according to HCA). On the other hand, samples with rosemary extract and thyme extract showed similarity to those with oregano extract and basil extract. For both meatballs and hamburgers, the taste desirability of the control samples showed the highest similarity to those with marjoram extract and lovage extract.

The consumers also assessed the overall consumer desirability of both meat products. For meatballs (Figure 1c), a high similarity was observed between samples with lovage extract and marjoram extract, as they were arranged in one cluster. Meatballs with oregano extract and basil extract formed the second cluster of products with the overall desirability to the consumers closest to the overall desirability of the control sample. Samples with thyme extract and rosemary extract formed a separate cluster, which had the weakest association with the others. Similar clusters were arranged in the case of overall desirability of hamburgers (Figure 2c) to the consumers. The strongest similarity was found between the samples with marjoram extract and lovage extract, and the weakest similarity was between the control sample and all other spice extracts. The HCA analysis showed that the spice extract additions influenced the overall consumer desirability of the tested meat products, and this effect was stronger for the fried product (hamburgers) than the steamed one (meatballs).

### 3.2. Sensory Profiling Results

In the sensory profiling, the perception of the following descriptors was defined and determined: aroma (meat, peppery, fried, spicy, essential oil, and bitter) and taste (meat, salty, broth, fried, spicy, essential oil, and bitter). The results of the sensory profiling of the meatballs and hamburgers with spice ethanol extracts and the control sample (without any extract) are presented in Table 2.

Principal component analysis (PCA) was used to study the relations between the aroma and/or taste attributes characteristic sensory profiles of meat products (variables) and to derive factors according to which these variables can be classified. PCA was also used to map the variants tested in our experiment (i.e., samples with selected spice extracts) into these factors. The PCA showed that the first two factors (F1 and F2) were the most important elements explaining variation in the data. For meatballs, they explained approximately 71% of the total variance for aroma and 87% for taste. For hamburgers, those factors (F1 and F2) explained approximately 74% of the total variance for aroma and 82% for taste. Therefore, they were selected for data interpretation. It is worth highlighting that F1 dominated in the explanation of taste: it explained approximately 70% of the taste variance.

The absolute values of the factor coordinates of the variables show the relationship between the factors and the sensory attributes (Table 3). For the aroma attributes of both meat products, the first factor (F1) was most strongly related to meat, spicy and essential oil aromas, the second factor (F2) to the bitter aroma. For taste attributes of meatballs and hamburgers, F1, the most strongly related to the meat, salty, broth, essential oil, and spicy taste, and F2 to the fried taste.

**Figure 3:**
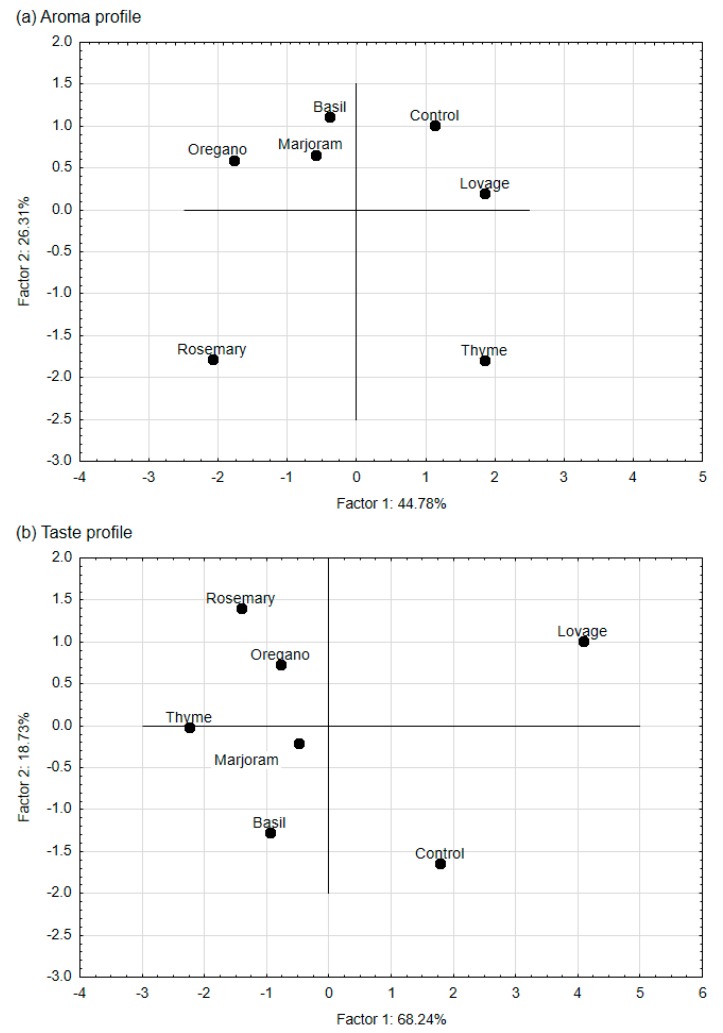
Map of the variants of pork meatballs with the addition of ethanol extracts of the spices (rosemary, thyme, lovage, marjoram, basil, oregano; 0.05%) and the control sample (without any extract) into factors (F1 × F2). Case–factor coordinate plots based on the attributes of (**a**) aroma profiles and (**b**) taste profiles (PCA analysis).

**Figure 4:**
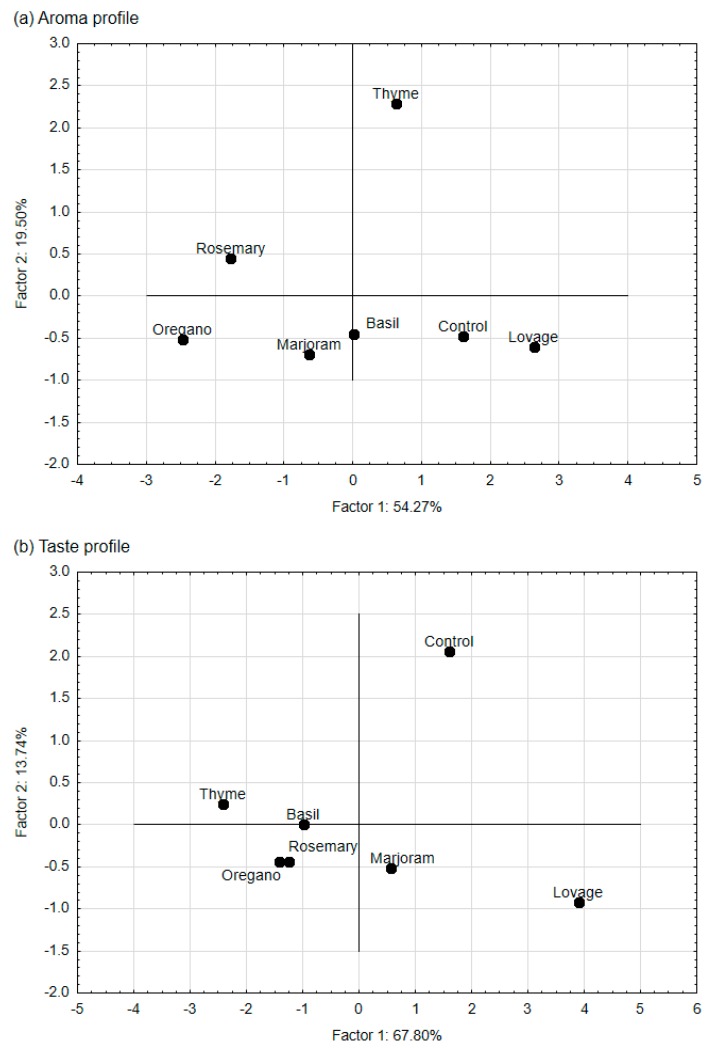
Map of the variants of pork hamburgers with the addition of ethanol extracts of the spices (rosemary, thyme, lovage, marjoram, basil, oregano; 0.05%) and the control sample (without any extract) into factors (F1 × F2). Case–factor coordinate plots based on the attributes of (**a**) aroma profiles and (**b**) taste profiles (PCA analysis).

In the case of meatball and hamburger aroma profiles (case-factor coordinate plots—Figure 3a and Figure 4a, Table 2), the control samples and the samples with lovage extract (which were characterised by a low intensity of spicy, essential oil, and bitter aromas) were plotted close together to the right side of the F1 axis. The samples with thyme, whose aroma profile had a low intensity of spice and essential oil aroma but a higher intensity of bitter aroma compared with the control sample, was also placed to this side of the F1 axis but the opposite side of the F2 axis. On the left side of the F1 axis were placed samples with rosemary extract (with a higher intensity of the essential oil aroma and spicy aroma compared with the control sample), samples with basil extract and marjoram extract (with a higher intensity of the spicy aroma than the control sample and low essential oil aroma), and those with oregano extract (with high intensity of spicy aroma).

For taste descriptors, the projection of the meatball variants on the factor-plane F1 × F2 (Figure 3b) shows that the control samples and samples with lovage extract were plotted to the right side of the F1 axis (i.e., they have positive coordinate values for F1). Meatballs with lovage, which were characterised by a low intensity of the spicy, essential oil and bitter tastes and a high intensity of broth taste, had a taste profile similar to the control sample (the differences between mean intensity of the descriptors for the sample with lovage and control were insignificant; Table 2). Samples with other spice extracts were plotted to the left side (negative coordinate values for F1). Their taste profiles were characterised by a higher intensity of the spicy and essential oil tastes (samples with rosemary extract and thyme extract) and/or the bitter taste (those with thyme, basil, and oregano extract) than the control sample. For hamburgers, the mapping of the samples based on their taste profiles (Figure 4b) shows that the control samples and the samples with lovage and marjoram extract were placed on the right side of the F1. In contrast, the other samples were placed on the left side—the taste profile of those samples was characterised by a higher intensity of the essential oil and spicy tastes (samples with rosemary and thyme extracts) and the bitter taste (for thyme, basil and oregano extracts), and a lower intensity of the broth tastes compared with the control sample (Table 2).

## 4. Discussion

Due to fast changes in consumer expectations and demands, developing new products is a necessity for producers to survive on the competitive food market [41]. The participation of consumers in the initial phase of the new food product development is one of the vital factors determining success on the market [42]. According to Lord [43], the unsatisfied needs of consumers help to create opportunities for the development of new products. Consumer satisfaction, achieved through fulfilling sensory expectations, among others, increases the likelihood of a product’s marketability [34,44].

Various active compounds of spice origin and their extracts were investigated for antimicrobial and antioxidant properties [36,45] as well as health benefits [8]. Some studies on the quality and stability of meat products with spice extract (particularly rosemary extract) also investigated their overall sensory quality [25,46,47,48]. Sutha et al. [48] evaluated the effect of rosemary essential oil addition (0.05%, 0.10%, and 0.25%) on the physico-chemical (pH, emulsion stability, product yield, and shear force value) and sensory qualities of chicken nuggets. They found that the additions were accepted by the panellists. Manhani et al. [25] compared the antioxidant potential of rosemary and oregano deodorized commercial extracts (0.04% addition) in precooked beef hamburgers by assessing the changes in lipid oxidation (TBRS values) and sensory analysis (colour, taste, odour evaluation using a 9-point hedonic scale) during 30 days of frozen storage. They showed that the hamburgers with those extracts were characterized by higher oxidation stability and satisfactory sensory quality compared to the control at the end of storage time. They also reported that the sensory analysis did not allow establishing a correlation between the scores of sensory attributes and the chemical changes. However, there is not much research regarding the effect of these additives on the sensory profile of food meat products and its acceptance by consumers.

In our research, both consumer tests and sensory profiling concerning meat products with the addition of selected spice extracts were carried out. The results of sensory profiling (Table 2) helped to explain the desirability of meat products with the addition of the selected spice extracts to consumers. These results can be an indication of which of the attributes of taste and smell, introduced into the product together with spice extracts, satisfy consumers, and which should be levelled.

According to the HCA analyses concerning the results of consumer tests, the meat products with the addition of rosemary extract and thyme extract formed one group (Figure 1 and Figure 2). These products are characterised by a higher intensity of both the spice and essential oil tastes than the control sample, which influenced consumer acceptance of the products. Products with thyme extract were also characterised by a higher intensity of the bitter taste, but it was not the case in the profile of products with rosemary extracts. The higher intensity of the bitter taste in the products with thyme extract compared to the products with rosemary extract may be a result of the higher content of phenolic compounds in the thyme extract. The previous studies reported the higher total phenolic content in the case of thyme extract than rosemary extract [12]. Spicy tastes and aromas usually do not come from a single compound but result from the presence of various compounds, including terpenes, essential oils, and aldehydes in plant products and foods. Many of these substances are volatile, which gives them a potent fragrance [49]. Therefore, they have great potential in creating the smell and taste of products. The essential oil taste and smell probably come from essential oils present in a particular spice (e.g., thymol and carvacrol in thyme [50], and carnosol, rosmanol, and geraniol in rosemary [8]). Our research shows that the combination of these high-intensity attributes in the product profile diminished the consumer acceptance of the meat product.

Products with basil extract and oregano extract formed the second separate group based on the consumer test results. They were characterised by higher bitter tastes and spice aromas compared with the control sample. Phenolic compounds are responsible for the bitterness and astringency of many foods and beverages [51], and so are spices. The bitter taste can decrease the consumer acceptance of food products [52,53], particularly those for which this taste is not characteristic. Our study shows that some of the spice ethanol extracts are carriers of the bitter taste, and this can decrease the overall desirability of pork meatballs and hamburgers. Consumers can rate overall palatability without being consciously aware of all food ingredients, especially those present at near-threshold levels [54,55] which are characteristic for individuals [56]. For this reason, the masking of bitterness can be crucial in the development of new products with spice addition. On the other hand, not all consumers are averse to bitter tastes. The findings of the previous study reveal that females and respondents with higher education levels demonstrate a higher acceptance of bitter tastes, as well as specific individual characteristics, such as high compensatory health beliefs [57]. Perhaps these groups of consumers could be encouraged to buy meatballs and hamburgers with the addition of extracts from basil and oregano if the label highlighted their positive impact, e.g., on the oxidative stability of the product.

According to overall consumer desirability, the third group was formed by products with lovage extract and marjoram extract. The intensity of the essential oil and bitter aroma and bitter taste of the samples were not statistically different from the control sample (Table 2). The aroma and taste profile of meat products with lovage extract was the most similar to the control sample. It may result from a relatively low content of total phenolics in lovage extracts compared with other spice extracts, which was reported in the previous studies [36]. However, it is worth highlighting that the lovage extract was also a carrier of broth taste and had a positive effect on its intensity in the meat products compared with the other extracts. It seems to be important for explaining the consumer acceptance of products with lovage extract. Further studies are necessary to find a relationship between a particular compound/compounds of lovage extract and the broth taste intensity in meat products with its addition. A high consumer acceptance of meat products with marjoram extract, despite the high intensity of the spicy aroma, spicy taste, and essential oil taste in their sensory profile, may explain the popularity of this spice. Consumers who often use marjoram for seasoning dishes may get used to its characteristic aroma and flavour.

## 5. Conclusions

The sensory quality of foods is one of the main factors in the acceptance of a product on the market. For this reason, studying factors that influence consumer desirability is crucial in food product development. The study shows that ethanol extracts of spices allow for the creation of new meat products with high consumer desirability. However, the high intensity of essential oil and spicy tastes of the spice extracts might be a limitation on the application of the extracts.

## Figures and Tables

**Table 1:** Mean scores (n = 246) ± standard deviation of the desirability of pork meatballs and hamburgers with ethanol extracts of the spices (rosemary, thyme, lovage, marjoram, basil, oregano; 0.05%) and the control sample (without any extract) to the consumers.

Consumer Desirability	Addition of Spice Ethanol Extracts						
	Rosemary	Thyme	Lovage	Marjoram	Basil	Oregano	Control
Meatballs							
aroma	4.8 ± 0.98 ^a^	5.1 ± 1.00 ^a^	7.6 ± 0.98 ^b^	6.9 ± 0.98 ^ab^	6.4 ± 0.92 ^ab^	6.3 ± 0.92 ^a^	6.6 ± 0.88 ^ab^
taste	4.9 ± 0.94 ^a^	5.1 ± 0.95 ^a^	7.7 ± 0.96 ^b^	7.0 ± 0.96 ^ab^	5.7 ± 0.88 ^a^	5.8 ± 0.98 ^a^	6.5 ± 0.91 ^ab^
overall	4.9 ± 0.94 ^a^	5.1 ± 0.95 ^a^	7.7 ± 1.02 ^b^	7.0 ± 1.02 ^ab^	5.9 ± 0.94 ^ab^	5.8 ± 1.06 ^ab^	6.5 ± 0.91 ^ab^
Hamburgers							
aroma	5.2 ± 0.95 ^a^	5.1 ± 0.98 ^a^	7.6 ± 0.97 ^b^	6.9 ± 1.00 ^ab^	6.0 ± 0.89 ^ab^	5.6 ± 0.88 ^ab^	6.8 ± 1.00 ^ab^
taste	5.4 ± 0.94 ^a^	5.1 ± 0.95 ^a^	7.6 ± 1.00 ^b^	7.0 ± 0.98 ^ab^	6.0 ± 0.92 ^a^	5.7 ± 1.10 ^a^	6.9 ± 1.04 ^ab^
overall	4.9 ± 0.96 ^a^	5.1 ± 0.95 ^a^	7.7 ± 1.03 ^b^	7.0 ± 1.07 ^ab^	5.7 ± 0.97 ^ab^	5.8 ± 1.15 ^ab^	6.5 ± 1.01 ^ab^

Different letters in the same raw for particular products denote a significant difference at *p* < 0.05 (one-way ANOVA, and post hoc Tukey test).

**Table 2:** Mean scores (n = 8) of sensory taste and aroma profiling of pork meatballs and hamburgers with ethanol extracts of the spices (rosemary, thyme, lovage, marjoram, basil, oregano; 0.05%) and the control sample (without any extract).

Sensory Attributes	Addition of Spice Ethanol Extracts						
	Rosemary	Thyme	Lovage	Marjoram	Basil	Oregano	Control
Meatballs							
Aroma							
meat	5.0 ± 0.3 ^a^	5.6 ± 0.2 ^ba^	5.9 ± 0.3 ^b^	5.1 ± 0.4 ^a^	5.0 ± 0.4 ^a^	5.0 ± 0.4 ^a^	5.6 ± 0.4 ^ba^
peppery	0.8 ± 0.2 ^a^	0.6 ± 0.2 ^a^	0.7 ± 0.3 ^a^	0.8 ± 0.2 ^a^	0.8 ± 0.2 ^a^	1.0 ± 0.2 ^a^	1.0 ± 0.2 ^a^
fried	0.4 ± 0.3 ^a^	0.2 ± 0.2 ^a^	0.2 ± 0.2 ^a^	0.2 ± 0.1 ^a^	0.0 ± 0.0 ^a^	0.2 ± 0.1 ^a^	0.2 ± 0.2 ^a^
spicy	1.9 ± 0.2 ^bc^	0.8 ± 0.2 ^ab^	0.4 ± 0.2 ^a^	2.0 ± 0.4 ^bc^	1.7 ± 0.4 ^bc^	2.2 ± 0.3 ^c^	0.2 ± 0.1 ^a^
essential oil	2.8 ± 0.2 ^c^	0.2 ± 0.2 ^a^	0.2 ± 0.2 ^a^	0.2 ± 0.1 ^a^	0.7 ± 0.2 ^ab^	1.4 ± 0.2 ^bc^	0.0 ± 0.0 ^a^
bitter	0.4 ± 0.2 ^ab^	1.20 ± 0.3^b^	0.0 ± 0.0 ^a^	0.0 ± 0.0 ^a^	0.2 ± 0.2 ^a^	0.2 ± 0.2 ^a^	0.2 ± 0.1 ^a^
Taste							
meat	4.9 ± 0.3 ^a^	5.2 ± 0.3 ^a^	6.4 ± 0.3 ^b^	5.4 ± 0.3 ^a^	5.2 ± 0.3 ^a^	5.0 ± 0.3 ^a^	5.6 ± 0.3 ^ab^
salty	2.0 ± 0.2 ^a^	2.0 ± 0.2 ^a^	3.0 ± 0.3 ^a^	2.4 ± 0.3 ^a^	2.0 ± 0.2 ^a^	2.4 ± 0.2 ^a^	2.3 ± 0.3 ^a^
broth	1.9 ± 0.3 ^a^	1.8 ± 0.3 ^a^	3.5 ± 0.3^b^	2.0 ± 0.3 ^a^	2.3 ± 0.3 ^a^	1.9 ± 0.3 ^a^	2.8 ± 0.4 ^ab^
fried	0.1 ± 0.2 ^a^	0.2 ± 0.2 ^a^	0.0 ± 0.0 ^a^	0.2 ± 0.2 ^a^	0.2 ± 0.2 ^a^	0.1 ± 0.1 ^a^	0.2 ± 0.2 ^a^
spicy	4.9 ± 0.3 ^c^	5.0 ± 0.3 ^c^	2.8 ± 0.2 ^ab^	4.0 ± 0.2 ^bc^	3.8 ± 0.3 ^abc^	4.0 ± 0.3 ^bc^	2.0 ± 0.3 ^a^
essential oil	1.5 ± 0.3 ^b^	1.5 ± 0.2 ^b^	0.2 ± 0.2 ^a^	1.0 ± 0.3 ^ab^	0.6±0.2 ^a^	1.2 ± 0.4 ^b^	0.0 ± 0.1 ^a^
bitter	0.4 ± 0.2 ^a^	1.7 ± 0.3 ^b^	0.2 ± 0.2 ^a^	1.0 ± 0.2 ^ab^	1.8±0.2^b^	1.2 ± 0.3 ^b^	0.4 ± 0.2 ^a^
Hamburgers							
Aroma							
meat	5.7 ± 0.3 ^a^	6.1 ± 0.4 ^a^	6.9 ± 0.3 ^a^	5.8 ± 0.4 ^a^	6.2 ± 0.3 ^a^	5.7 ± 0.3 ^a^	6.7 ± 0.3 ^a^
peppery	3.4 ± 0.3 ^a^	3.4 ± 0.3 ^a^	3.6 ± 0.3 ^a^	3.3 ± 0.3 ^a^	3.2 ± 0.2 ^a^	3.0 ± 0.3 ^a^	3.4 ± 0.3 ^a^
fried	2.6 ± 0.3 ^a^	2.7 ± 0.3 ^a^	2.9 ± 0.3 ^a^	2.7 ± 0.3 ^a^	2.9 ± 0.2 ^a^	2.5 ± 0.2 ^a^	2.7 ± 0.2 ^a^
spicy	1.7 ± 0.3 ^b^	0.2 ± 0.2 ^a^	0.4 ± 0.2 ^a^	1.9 ± 0.2 ^b^	1.8 ± 0.2 ^b^	1.8 ± 0.2 ^b^	0.2 ± 0.2 ^a^
essential oil	2.2 ± 0.4 ^c^	0.4 ± 0.2 ^a^	0.2 ± 0.2 ^a^	0.2 ± 0.2 ^a^	0.5 ± 0.2 ^a^	1.2 ± 0.2 ^b^	0.0 ± 0.0 ^a^
bitter	0.5 ± 0.3 ^ab^	1.7 ± 0.3 ^b^	0.0 ± 0.0 ^a^	0.2 ± 0.2 ^a^	0.6 ± 0.3 ^ab^	0.2 ± 0.2 ^a^	0.0 ± 0.0 ^a^
Taste							
meat	5.7 ± 0.2 ^a^	6.1 ± 0.3 ^ab^	7.4 ± 0.3 ^b^	6.5 ± 0.3 ^a^	6.0 ± 0.2 ^ab^	5.8 ± 0.3 ^a^	6.5 ± 0.3 ^ab^
salty	3.1 ± 0.2 ^ab^	2.9 ± 0.2 ^a^	4.2 ± 0.2 ^b^	3.8 ± 0.3 ^ab^	2.9 ± 0.2 ^a^	3.1 ± 0.2 ^ab^	3.3 ± 0.3 ^ab^
broth	4.9 ± 0.3 ^a^	5.3 ± 0.3 ^a^	7.3 ± 0.3 ^b^	5.2 ± 0.3 ^a^	4.9 ± 0.2 ^a^	4.9 ± 0.3 ^a^	7.0 ± 0.2 ^b^
fried	0.5 ± 0.2 ^a^	0.2 ± 0.2 ^a^	0.9 ± 0.2 ^a^	0.5 ± 0.3 ^a^	0.5 ± 0.2 ^a^	0.5 ± 0.2 ^a^	0.2 ± 0.2 ^a^
spicy	4.7 ± 0.3 ^c^	4.9 ± 0.2 ^c^	2.9 ± 0.3 ^a^	3.6 ± 0.3 ^ab^	3.4 ± 0.3 ^ab^	4.2 ± 0.2 ^ab^	2.9 ± 0.3 ^a^
essential oil	1.2 ± 0.2 ^bc^	1.7 ± 0.2 ^c^	0.1 ± 0.1 ^a^	0.8 ± 0.2 ^b^	0.8 ± 0.2 ^b^	1.1 ± 0.3 ^bc^	0.1 ± 0.1 ^a^
bitter	0.2 ± 0.2 ^a^	1.4 ± 0.3^bc^	0.2 ± 0.2 ^a^	0.7 ± 0.2 ^ab^	1.5 ± 0.3 ^c^	1.2 ± 0.3^b^	0.2 ± 0.1 ^a^

Different letters in the same raw for particular products denote a significant difference at *p* < 0.05 (one-way ANOVA, and *post hoc* Tukey test).

**Table 3:** The factor loadings for the aroma and taste attributes of pork meatballs and hamburgers with ethanol extracts of spices (rosemary, thyme, lovage, marjoram, basil, oregano; 0.05%) and the control sample (without any extract).

Sensory Attributes	Hamburgers		Meatballs	
	F1	F2	F1	F2
Aroma				
meat	0.948 *	−0.185	0.918 *	−0.082
peppery	−0.770 *	0.198	−0.475	0.560
fried	0.759	−0.164	−0.300	−0.665
spicy	−0.891 *	−0.408	−0.892 *	−0.001
essential oil	−0.738 *	0.159	−0.804 *	−0.467
bitter	−0.066	0.937 *	0.291	−0.744 *
Taste				
meat	0.917 *	−0.078	0.921 *	0.078
salty	0.884 *	−0.347	0.871 *	0.347
broth	0.873 *	−0.336	0.967 *	−0.091
fried	0.585	0.760 *	−0.560	−0.767 *
spicy	−0.845 *	0.255	−0.835 *	0.482
essential oil	−0.913 *	0.258	−0.839 *	0.525
bitter	−0.688	0.110	−0.704	−0.349

Two factors (F1 and F2) were extracted by applying Principal Component Analysis (PCA) on the mean values of descriptive sensory scores. Sensory attributes with numbers marked * are believed to be most important.
